# Aureonitol, a Fungi-Derived Tetrahydrofuran, Inhibits Influenza Replication by Targeting Its Surface Glycoprotein Hemagglutinin

**DOI:** 10.1371/journal.pone.0139236

**Published:** 2015-10-13

**Authors:** Carolina Q. Sacramento, Andressa Marttorelli, Natalia Fintelman-Rodrigues, Caroline S. de Freitas, Gabrielle R. de Melo, Marco E. N. Rocha, Carlos R. Kaiser, Katia F. Rodrigues, Gisela L. da Costa, Cristiane M. Alves, Osvaldo Santos-Filho, Jussara P. Barbosa, Thiago Moreno L. Souza

**Affiliations:** 1 Laboratório de Vírus Respiratórios, Instituto Oswaldo Cruz, Fundação Oswaldo Cruz, Rio de Janeiro, Rio de Janeiro, Brazil; 2 Laboratório de Imunofarmacologia, Instituto Oswaldo Cruz, Fundação Oswaldo Cruz, Rio de Janeiro, Rio de Janeiro, Brazil; 3 Centro de Desenvolvimento Tecnológico em Saúde, Fundação Oswaldo Cruz, Rio de Janeiro, Rio de Janeiro, Brazil; 4 Laboratório de Química de Produtos Naturais 5, Farmanguinhos, Fundação Oswaldo Cruz, Rio de Janeiro, Rio de Janeiro, Brazil; 5 Instituto de Química, Universidade Federal do Rio de Janeiro, Rio de Janeiro, Rio de Janeiro, Brazil; 6 Laboratório de Taxonomia, Bioquímica e Bioprospecção de Fungos, Instituto Oswaldo Cruz, Fundação Oswaldo Cruz, Rio de Janeiro, Rio de Janeiro, Brazil; The University of Chicago, UNITED STATES

## Abstract

The influenza virus causes acute respiratory infections, leading to high morbidity and mortality in groups of patients at higher risk. Antiviral drugs represent the first line of defense against influenza, both for seasonal infections and pandemic outbreaks. Two main classes of drugs against influenza are in clinical use: M2-channel blockers and neuraminidase inhibitors. Nevertheless, because influenza strains that are resistant to these antivirals have been described, the search for novel compounds with different mechanisms of action is necessary. Here, we investigated the anti-influenza activity of a fungi-derived natural product, aureonitol. This compound inhibited influenza A and B virus replication. This compound was more effective against influenza A(H3N2), with an EC_50_ of 100 nM. Aureonitol cytoxicity was also very low, with a CC_50_ value of 1426 μM. Aureonitol inhibited influenza hemagglutination and, consequently, significantly impaired virus adsorption. Molecular modeling studies revealed that aureonitol docked in the sialic acid binding site of hemagglutinin, forming hydrogen bonds with highly conserved residues. Altogether, our results indicate that the chemical structure of aureonitol is promising for future anti-influenza drug design.

## Introduction

Acute respiratory infections are a major cause of morbidity and mortality and, therefore, have a great impact on public health [1]. Episodes of severe acute respiratory infections (SARI) are likely to be triggered by the influenza virus [2, 3]. Influenza is a zoonotic agent that can cause seasonal infections and pandemic outbreaks in humans [3]. The influenza virus has a negative-sense segmented RNA, a characteristic of members of the orthomyxovirus family [2]. To enter host cells, the influenza surface glycoprotein hemagglutinin (HA) binds to sialic acid residues on proteins localized in the cellular plasma membrane. Subsequently, virions are endocytosed, and the viral envelope and endocytic membrane are fused due to influenza protein M2 proton channel activity [4]. Viral ribonucleoproteins (RNP) are then released into the cytoplasm and transported to the cell nucleus, where transcription and replication of the viral genome occur. Following replication assembled virus particles bud through the cellular plasma membrane and are released via viral neuraminidase (NA) activity [5].

Although anti-influenza vaccines exist, several limitations make eradication through vaccination a difficult strategy. First, influenza has multiple zoonotic hosts [6]. The time frame to produce vaccines against novel influenza viruses is also generally very long [7]. The high cost of vaccine production and the fact that vaccines are only recommended for patient groups who are at high risk for serious illnesses are additional limitations [7].

Due to the limitations of vaccination, antiviral drugs are an important option for controlling influenza virus replication [8, 9]. Because the antigenic characteristics of viral strains that might cause future pandemic outbreaks are unpredictable, the stockpiling of anti-influenza drugs is a key step in pandemic preparedness [8, 9]. However, antiviral resistance to the adamantanes, M2-channel blockers, is very common. Neuraminidase inhibitors (NAIs) are the main class of antiviral drugs currently in clinical use [10], but mutants that are resistant and have decreased sensitivity to oseltamivir (OST) have been described [11, 12]. Therefore, the identification of molecules that can inhibit influenza strains resistant to these antivirals and/or block another step in the virus life cycle is necessary.

*Chaetomium* Kuntze ex Fries (*Chaetomiaceae*) is a cosmopolitan fungus found in soil and cellulose-containing substrates [13]. Members of this genus are rich sources of bioactive secondary metabolites with different chemical structures [14–16], such as alkaloids [17, 18], esters [19] and polyketides [20]. Among the secondary metabolites produced by this genus, aureonitol, a tetrahydrofuran (THF) derivative [21], is an abundant metabolite. Using the fungus *Chaetomium globosum* as a model organism, it has been shown that aureonitol acts as a transcriptional regulator for the synthesis of other secondary metabolites in this species [22]. Aureonitol has been isolated from different species of the genus *Chaetomium*, from pure cultures *in vitro* and in association with the plant *Helichrysum aureonitens* in nature [21]. Although it has been demonstrated that other THF derivatives are endowed with antiviral activity [23–25], including against influenza [26], the effects of aureonitol on influenza replication have not been characterized. We show here that aureonitol inhibits influenza replication by targeting conserved residues on HA.

## Materials and Methods

### Compound

**Fig 1:**
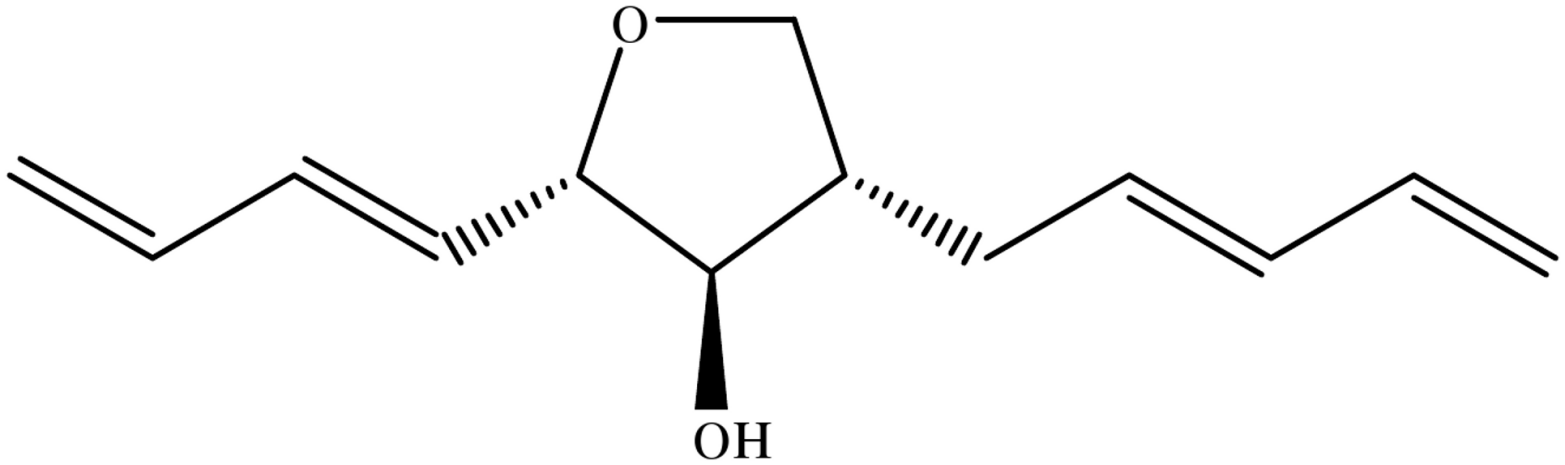
Chemical structure of aureonitol.

The THF derivative aureonitol (Fig 1) was isolated from mycelium plugs obtained from *in vitro* cultures of the fungus *Chaetomium coarctatum* and identified as previously described [21, 27–30]. A voucher of the specimen was deposited on the Filamentous fungal collection (IOC-FIOCRUZ; CCFF/IOC-4613). Aureonitol, at over 99% purity, was diluted in 100% dimethyl sulfoxide (DMSO) and stored at– 20°C. The resulting DMSO concentrations during the assays were below 0.1%, a level that is not significantly cytotoxic.

### Cells and viruses

Madin-Darby Canine Kidney cells (MDCK), donated by Influenza Reagent Resource (IRR; http://www.influenzareagentresource.org/) to the Brazilian National Influenza Center (NIC), were cultured with Minimum Essential Eagle*’s* Medium (MEM; LGC Biotecnologia, São Paulo, Brazil). Human embryonic kidney 293 cells (HEK293) were cultured with Minimum Dulbecco´s Modified Eagle Medium (DMEM; LGC Biotecnologia, São Paulo, Brazil) and used in transfection assays. These culture were supplemented with 10% fetal bovine serum (FBS; HyClone, Logan, UT, USA), 100 U/mL penicillin and 100 mg/mL streptomycin (Sigma-Aldrich). The cells were cultured at 37°C in a 5% CO_2_ atmosphere. A list of all the influenza A and B strains used in this study is presented in the supporting information file (Table A in S1 File). Experiments were conducted using either a laboratory-adapted strain or clinical isolates (Table A in S1 File). These viruses were grown in MDCK cells [31]. Viral stocks were aliquoted and stored at -70°C for further studies.

### Cell viability

Confluent (2 x 10^4^ cell/well) or semi-confluent (2 x 10^3^ cell/well) monolayers of MDCKs in 96-well culture plates were incubated with the compounds at different concentrations for 72 h. Then, 2,3-Bis-(2-Methoxy-4-Nitro-5-Sulfophenyl)-2*H*-Tetrazolium-5-Carboxanilide (XTT) at 5 mg/ml was added to the DMEM in the presence of 0.01% N-methyl-dibenzopirazina methyl sulfate (PMS). After incubation for 4 h at 37°C, the plates were read in a spectrophotometer at 492 nm and 620 nm [32]. The 50% cytotoxic concentration (CC_50_) was calculated by performing a linear regression analysis on the dose—response curves generated from the data.

### Yield reduction assay

Monolayers of MDCK cells (2 x 10^5^ cell/well) in 24-well plates were infected with influenza at different MOIs for 1 h at 37°C. The cells were then washed to remove residual viruses and varying concentrations of the compounds were added. At different time points after infection, viruses in the supernatant were harvested and titrated by end-point 50% cell culture infective dose (TCID_50_/mL) using MDCK cells (5x10^4^ cells/well in 96-well plates) [33, 34]. For comparison, the reference compound oseltamivir carboxylate (OST; kindly donated by Hoffman-La Roche Inc., Basel, Switzerland) was used as a control. Non-linear regression of the dose-response curves was performed to determine the 50%, 90% and 99% inhibitory effects on viral replication (EC_50_, EC_90_ and EC_99,_ respectively) for the tested and reference compounds.

### Measurements of influenza attachment/entry

Monolayers of 2 x 10^6^ cells in 6-well plates were incubated with different concentrations of aureonitol in the presence of influenza (MOI = 1) for 1 h at 4°C, a condition that allows only virus adsorption. Then, the cultures were washed with PBS, lysed with buffer A (10 mM HEPES, 1.5 mM MgCl_2_, 10 mMKCl, 0.5% NP-40, 1 mM DTT, 0.5 mM PMSF) and centrifuged (10 min. at 1000 x *g*). The resulting supernatant, which contained the plasma membrane, was submitted for quantitative RT-PCR to detect virus genome from particles that were able to adsorb onto the cells [35]. Quantitative RT-PCR to detect influenza A was performed based on the standard curve method described elsewhere [35]. A plasmid containing the influenza gene M1 was used as the reference. Notably, controls to verify the purity of the RNA preparations were performed to detect the housekeeping transcripts GAPDH, RNAse P, β-actin and long non-coding RNA (lncRNA). As expected, the supernatants were free of the nuclear exclusive transcript lncRNA (Fig A in S1 File) [35, 36].

Alternatively, HEK293 were transfected using lipofectamine 2000® with pCDNA 3.1 (+) containing the influenza HA insert between HindIII and XhohI sites (encoding for either HA types H1, H3 or B of the viruses shown in Table A in S1 File). These cells were also co-transfected with a retrovirus expressing the vector plasmid pNL4-3.Luc.R-.E- (NIH AIDS Reagent Program; https://www.aidsreagent.org/index.cfm). This last plasmid encodes for luciferase and HIV-1 proteins, except for those associated in the envelope, and Vpr. At 24 h after co-transfection, the cells were treated with 50 U/mL of NA from *Clostridium perfinges* (Sigma-Aldrich) to release tethered pseudotyped viruses. Next, pseudotyped viruses were concentrated under centrifugation with Centricon membranes to remove particles < 100 kDa. These viruses were quantified by ELISA against the retroviral antigen (Ag) p24 (Zeptometrix). Infection assays with pseudotyped viruses were performed with doses equivalent to 10 ng/ml of p24 Ag.

To evaluate the effects of aureonitol on influenza attachment/entry, we tested the following three different approaches with pseudotyped viruses: cells and pseudotyped viruses were treated simultaneously (a), cells were treated prior to infection with a pseudotyped virus (b), or pseudotyped viruses were treated with the compound prior to cellular infection (c). In protocol (a), MDCKs (10^5^ cell/well) in 24-well plates were exposed to the pseudotypes virus (10 ng/ml of p24 Ag) and simultaneously treated with different concentrations of aureonitol for 1 h at 4°C; under these conditions, viruses bind but are not able to enter the cells. Next, the cells were washed with PBS, covered with DMEM containing 5% FBS, and the temperature was raised to 37°C for 3 h [8]. After this period, the cells were lysed and luciferase activity was measured with Promega’s kit, according to manufacturer’s instructions. When we raised the temperature to 37°C, only the bound virus particles were able to penetrate and replicate; however, if aureonitol blocked influenza attachment/entry, less virus would enter, and consequently the luciferase activity would be reduced at 3 h after infection. In protocol (b), the same number of cells were pre-treated for 1 h at 37°C with aureonitol at different concentrations and then washed with PBS. The cells were subsequently exposed to pseudotyped virus for 1 h at 4°C, washed with PBS again, and then incubated in DMEM with 5% FBS for 3 h. Finally, the cells were lysed, and luciferase activity was measured, as described above. In protocol (c), different concentrations of aureonitol were prepared, and these solutions were diluted in PBS containing pseudotyped viruses. These solutions were incubated for 1 h at room temperature, after which the appropriate dilutions were added to monolayers of MDCKs for 1 h to infect the cells with virus. After infection, the cells were washed with PBS and incubated in DMEM with 5% FBS for 3 h. The cells were then lysed and the virus was tittered, as in protocol (a). For comparison, we also employed protocols (a), (b) and (c) to analyze the effects of OST and specific anti-sera as negative and positive controls, respectively.

### NA inhibition assay

To evaluate the ability of the compound to inhibit influenza NA activity, we performed cell-free assays using the NA-Star kit (Life Technologies, CA) according to the manufacturer’s instructions. Briefly, influenza NA activity was titrated and then measured in the presence of different concentrations of the compound to quantify enzyme inhibition. The concentration able to inhibit 50% of influenza`s NA activity (IC_50_) was calculated using a non-linear regression. For comparison, every assay was performed with OST carboxylate as a positive control.

### HA inhibition assay

Hemagglutination inhibition assays (HAI) were performed according to the protocol recommended by the World Health Organization (WHO) [37,38]. Briefly, aureonitol or the reference sera against the influenza virus (CDC/Atlanta; WHO Collaborating Center for influenza), were treated with receptor destroying enzyme (RDE; Denka-Seiken, Japan) to inhibit nonspecific hemagglutination inhibitors. These sera were then incubated with 4 HAU of influenza HA along with guinea pig red blood cells (RBC) at a concentration of 0.5% for 1 h. After this incubation, HAI was read. The results are expressed as the reciprocal of the highest dilution that inhibited hemagglutination for the control sera and as the minimal inhibitory concentration (MIC) for aureonitol.

### Molecular studies of aureonitol`s docking site

To perform *in silico* studies, the structure of aureonitol was designed using Accelrys Draw 4.1 software (Accelrys, Inc), hydrogens were added and the geometry was optimized. Aureonitol was docked into different regions of HA, encompassing the entire protein structure. Alternatively, aureonitol was docked only in predetermined active sites of HA. To perform these experiments, we used the "Dock a Ligand" option in Arguslab 4.0.1 (Planaria Software LLC)[39]. The crystal structures of HA proteins representative of the strains used in this study were also analyzed (Table B in S1 File). These structures are available from the Protein Data Bank (PDB; http://www.rcsb.org) [40]. A spacing of 0.4 Å between the grid points was used. The ligand was assumed to be flexible while the protein was assumed to be rigid. A maximum of 150 poses were analyzed, and each docking run was repeated three times to obtain the best results. The binding site box was set to 25 × 25 × 25 Å to encompass the entire active site of the enzyme. The results were analyzed using PoseView software[41,42].

### Statistical analysis

The dose-response curves used to calculate the pharmacological parameter values were generated using Excel for Windows [42]. When appropriate, Student`s-t-test was used to evaluate significant differences, with P < 0.05 set as the threshold for significance. All of the experiments were performed at least three times, and the results are displayed as the mean ± standard error of the mean (SEM).

## Results

### Aureonitol inhibits influenza replication in a dose-, MOI- and time-dependent fashion

Because aureonitol is a THF derivative, and other compounds with this basic chemical structure may possess antiviral activity, we evaluated aureonitol’s ability to inhibit influenza replication. Aureonitol inhibited influenza replication 24 h after infection with the A(H3N2) subtype in a dose-dependent fashion, with EC_50_ values of 30, 100 and 183 nM at MOIs of 0.01, 0.05 and 0.01, respectively (Table 1, Figs B-D in S1 File). At 48 h after infection, aureonitol's potency was slightly reduced, as the obtained EC_50_ values were 48, 121 and 201 nM for MOIs of 0.01, 0.05 and 0.1, respectively (Table 1, Figs B-D in S1 File). For comparison, at 24 post-infection OST presented EC_50_ values of 12, 30 and 49 nM for MOIs of 0.01, 0.05 and 0.1, respectively (Table 1, Figs B-D in S1 File). At 48 h after infection, the EC_50_ concentration for OST also increased slightly to 28, 38 and 56 nM for MOIs of 0.01, 0.05 and 0.1, respectively (Table 1, Figs B-D in S1 File). Similar observations were also made based on the pharmacological parameters of antiviral effect 90 and 99% (Table C in S1 File). Together, these results indicate that, although OST is more potent than aureonitol, both compounds act at nanomolar concentrations and are potent against influenza replication *in vitro*.

**Table 1:** MOI- and time-dependent inhibition of influenza replication by aureonitol.

	EC_50_ (nM)			
	24 h		48 h	
MOIs	Aureonitol	OST	Aureonitol	OST
0.1	183 ± 21	49 ± 2.6	201 ± 13	56 ± 3.1
0.05	100 ± 16	30 ± 2.3	121 ± 18	38 ± 2.8
0.01	30 ± 3.4	12 ± 0.9	42 ± 3.9	28 ± 1.1

Next, we evaluated aureonitol's efficacy against circulating strains of influenza A and B. Aureonitol inhibited the replication of the laboratory-adapted and clinically isolated strains of influenza A(H3N2) with similar efficiencies, although it was three times more potent against the laboratory-adapted virus (Table 2, Table D and Fig E-H in S1 File). Similarly, aureonitol also inhibited Influenza A(H1N1)pdm09 replication with an efficiency that was not significantly different than that observed for the influenza A(H3N2) viral strains (Table 2, Table D and Figs E-H in S1 File). Again, the doses to inhibit replication of the clinical isolates by 50% were four-times higher than the EC_50_ for the laboratory-adapted strain (Table 2, Table D and Figs E-H in S1 File). Although inhibition of influenza B replication was achieved with aureonitol, the pharmacological parameters for potency and efficiency were higher than those observed for the influenza A subtypes (Table 2, Table D and Figs E-H in S1 File). As a control, OST was used in all experiments (Table 2, Table D and Figs E-H in S1 File). The reference compound was more potent and efficient than aureonitol by 10- and 100-times, respectively. Nevertheless, our data indicate that aureonitol's chemical structure is promising for the future development of novel influenza antivirals.

**Table 2:** Antiviral activity of Aureonitol against influenza A and B viruses.

Influenza Type (subtypes)	EC_50_ (nM)		EC_90_ (nM)		EC_99_ (nM)	
	Aureonitol	OST	Aureonitol	OST	Aureonitol	OST
**A(H3N2)** *	100 ± 16	30 ± 2.3	3000 ± 236	90 ± 14	3091 ± 168	98 ± 8
**A(H3N2)**	312 ± 23	32 ± 2.1	2801 ± 123	92 ± 16	2912 ± 322	112 ± 13
**A(H1N1)pdm09**	417 ± 30	12 ± 1.9	2008 ± 212	78 ± 9	2023 ± 113	92 ± 7
**B**	2012 ± 87	52 ± 7.1	15876 ± 432	132 ± 25	16789 ± 455	155 ± 18

*Laboratory-adapted influenza A(H3N2).

### Aureonitol is safe to be used *in vitro*

**Fig 2:**
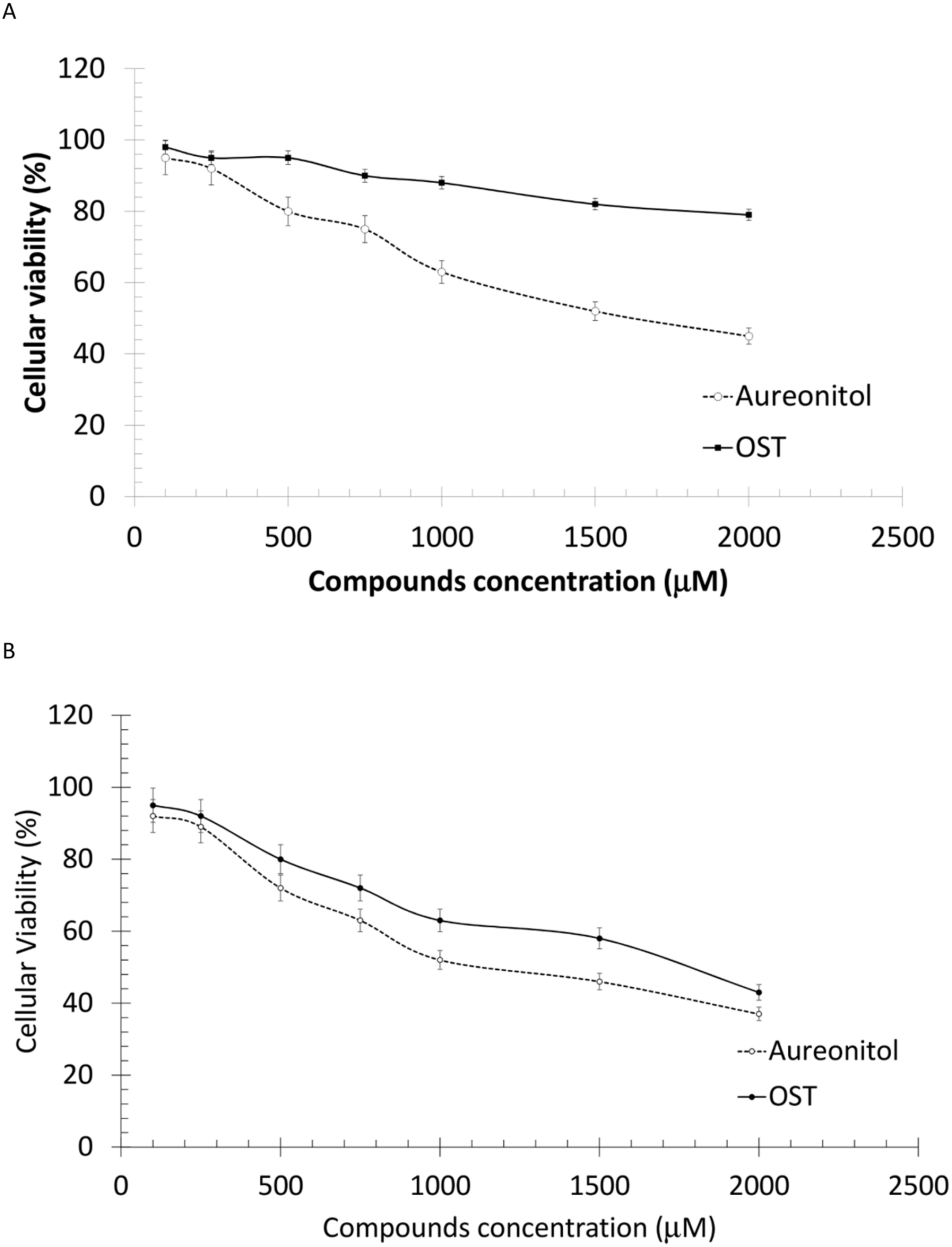
Cytotoxicity of aureonitol. MDCKs were seeded at full confluence (2 x 10^4^ cells) (A) or semi-confluence (2 x 10^3^ cells) (B) in 96-well plates. The cells were then incubated with the indicated concentrations of the compound for 72 h, after which XTT (5 mg/ml) and PMS (0.01%) were added. After 4 h of incubation, the plates were read at 492 nm and 620 nm. Experiments from both panels were performed 5 times in triplicate.

Aureonitol had very low cytotoxicity, similar to OST; these compounds had CC_50_ values of 1426 ± 13 μM and 2132 ± 26 μM, respectively (Fig 2A) when tested in cell cultures that were 100% confluent. When the cytotoxicity was tested at 60% confluency, the CC_50_ values decreased to 1357 ± 19 μM for aureonitol and 1683 ± 32 μM for OST (Fig 2B). The selectivity index (SI), which is the ratio between the CC_50_ and EC_50_ values, for aureonitol and OST varied due to the different potencies of these drugs against the influenza viruses used (Table 3). Although OST's SI value was higher than that observed for aureonitol, our molecule’s SI value indicates that it is still very safe to be used *in vitro*, as the threshold of cytotoxicity is thousands of times higher than the antiviral potency (Table 3).

**Table 3:** Selectivity index of Aureonitol for Influenza A and B viruses.

Influenza types (subtypes)	EC50 (μM)		CC50 (μM)		SI	
	Aureonitol	OST	Aureonitol	OST	Aureonitol	OST
**A(H3N2)** *	0.100	0.030	1426	2132	14260	71067
**A(H3N2)**	0.312	0.032	1426	2132	4571	66625
**A(H1N1)**	0.417	0.012	1426	2132	3420	177667
**B**	2.012	0.052	1426	2132	709	41000

*Laboratory-adapted influenza A(H3N2).

### Aureonitol inhibits hemagglutination, but not NA activity, and consequently impairs influenza entry

Influenza surface glycoproteins HA and NA are responsible for viral entry and release, respectively [43]. Moreover, these proteins are validated anti-influenza targets [44, 45]. Aureonitol inhibited hemagglutination with MICs ranging from 60 to 200 nM (Table 4) for the various influenza A and B strains used. As a control, standard serum raised in sheep against the different influenza types and subtypes were tested and inhibited hemagglutination with appropriate dilution (Table 4). The correlation between aureonitol’s potency over hemagglutination and viral replication inhibition was comparable (i.e., both pharmacological parameters revealed potencies in the nanomolar range). This good correlation could indicate that hemagglutination inhibition is aureonitol’s target on the influenza life cycle [46, 47].

**Table 4:** Anti-hemagglutination activity of Aureonitol against influenza A and B.

Influenza types (subtypes)	Minimal inhibitory concentration or reverse dilution	
	Aureonitol (nM)	Specific anti-sera
**A(H3N2)** *	100	1024
**A(H3N2)**	120	512
**A(H1N1)**	60	1024
**B**	400	512

*Laboratory-adapted influenza A(H3N2).

**Fig 3:**
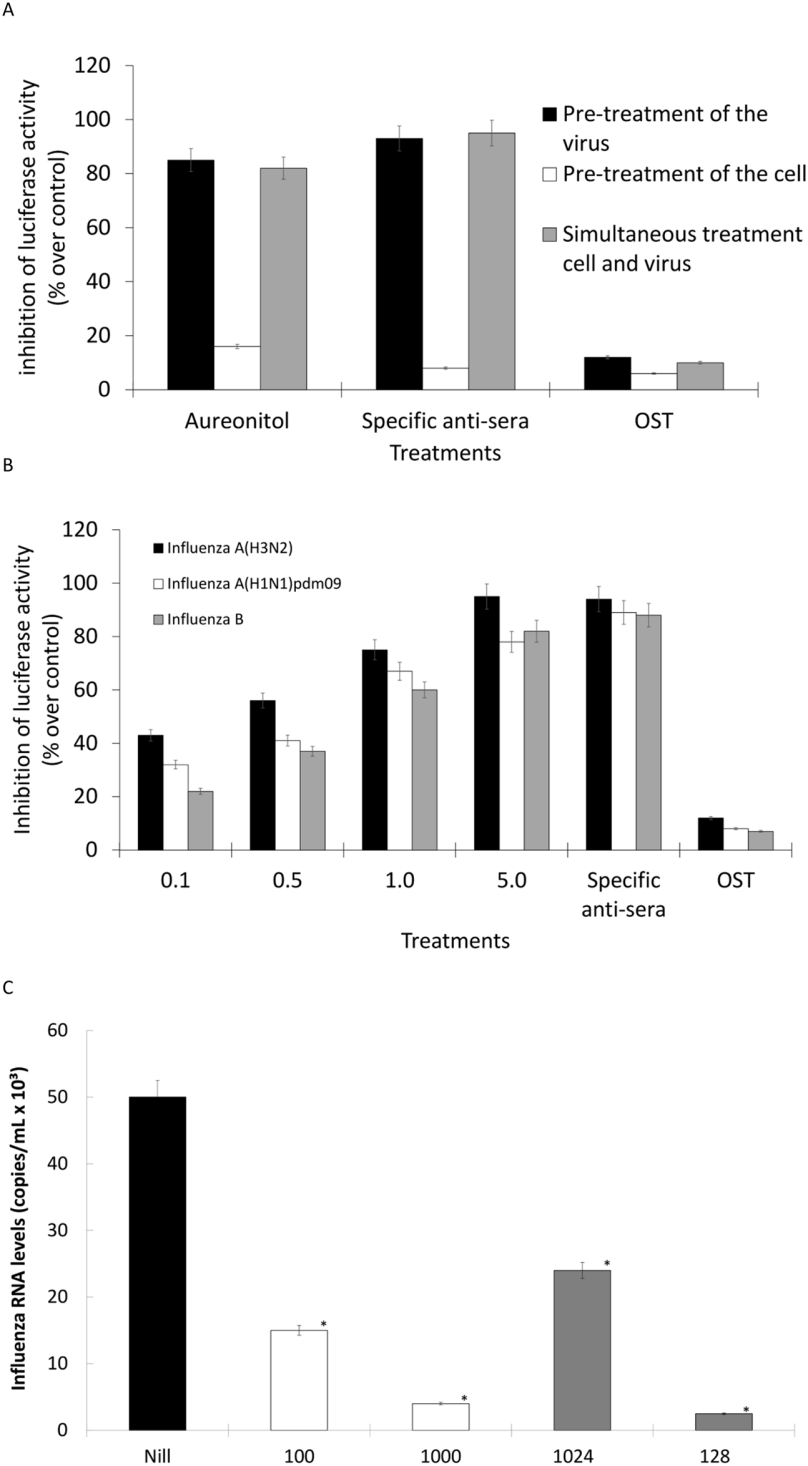
Effects of aureonitol on influenza attachment/entry. (A) Virus particles were pre-treated for 1 h at room temperature. This mixture was diluted and incubated with MDCK cells for 1 h at 37°C (black bar). MDCK cells were pre-treated for 1 h at 37°C and washed. The cells were then exposed to pseudotyped influenza for 1 h at 4°C, washed again, and the temperature was shifted to 37°C (white bar). MDCK cells were exposed to the indicated influenza pseudotyped virus and treated for 1 h at 4°C. The cells were then washed with PBS and the temperature was raised to 37°C (gray bars). (B) Using the last treatment approach, pseudotyped influenza viruses representative of the indicated strains were used, and different concentrations of aureonitol were evaluated. (C) MDCKs (2 x 10^6^ cells) were infected with influenza at a MOI of 1.0 for 1 h at 4°C in the presence of the indicated treatments. After that, the cells were washed, lysed with buffer A and centrifuged (10 min. at 1000 x *g*). RNA was extract from the supernatant fraction, and quantitative RT-PCR to detect influenza A genome was performed. In panels A and B, luciferase activity was measured with a commercial kit (Promega). In panel C, the standard curve method was employed using a plasmid containing the influenza M1 gene as a reference. The experiments were performed 4 times. *P < 0.01.

To monitor the anti-hemagglutination activity of aureonitol in a functional way, we performed a series of adsorption inhibition assays. From the results, it is apparent that aureonitol inhibited influenza attachment/entry either when the viruses were pretreated with the compound or when the viruses and cells were treated simultaneously (Fig 3A). On the other hand, pre-treatment of the cells had no effect on the inhibition of influenza attachment/entry (Fig 3B). These results suggest that aureonitol targets a viral, rather than a cellular structure, with primary importance to attachment/entry. This finding is consistent with the hemagglutination inhibition promoted by our compound. As a positive control, specific sera against the influenza A(H3N2) pseudotyped virus displayed the same results as aureonitol (Fig 3A). The negative control, OST had no relevant effect on influenza attachment/entry (Fig 3A). Aureonitol is able to inhibit influenza attachment/entry for different subtypes of influenza A and influenza B to different degrees (Fig 3B). Finally, we evaluated whether our compound could decrease influenza attachment onto cells, using virus infectivity assays with real rather than chimeric viruses. Indeed, aureonitol significantly blocked influenza adsorption at both suboptimal and optimal concentrations (Fig 3C). These results confirm that aureonitol is endowed with a mechanism of action that is different from most anti-influenza drugs in clinical use.

**Fig 4:**
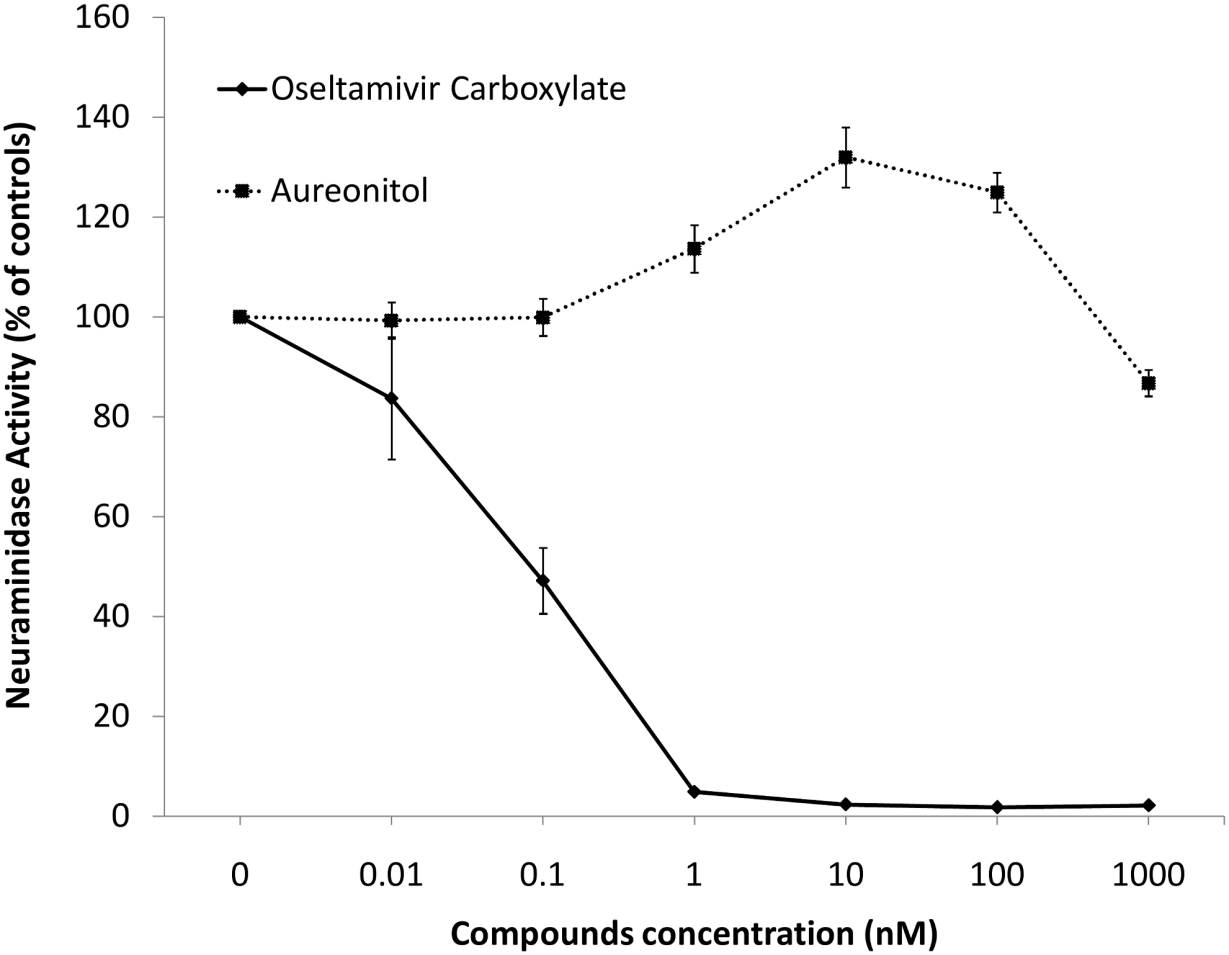
Aureonitol does not inhibit influenza NA activity. The NA activity of the influenza A H3N2 virus was measured in the presence of the indicated concentrations of aureonitol or the reference compound OST-c using a chemiluminescent substrate, NA-star kit (Applied Biosystems, CA). The results were obtained in relative luminescence units (RLU) but were converted to a percentage of the control for normalization of the data displayed. This experiment was performed 4 times.

Nevertheless, we tested whether aureonitol inhibits influenza NA activity because another THF derivative is known to have such an activity [26]. Aureonitol had no effect on this enzyme activity (Fig 4), even when tested at 1000 nM (10 times its EC_50_). As a control, OST inhibited NA activity with an IC_50_ value of 0.1 ± 0.012 nM (Fig 4).

### Aureonitol docked on conserved amino acid residues on HA

**Fig 5:**
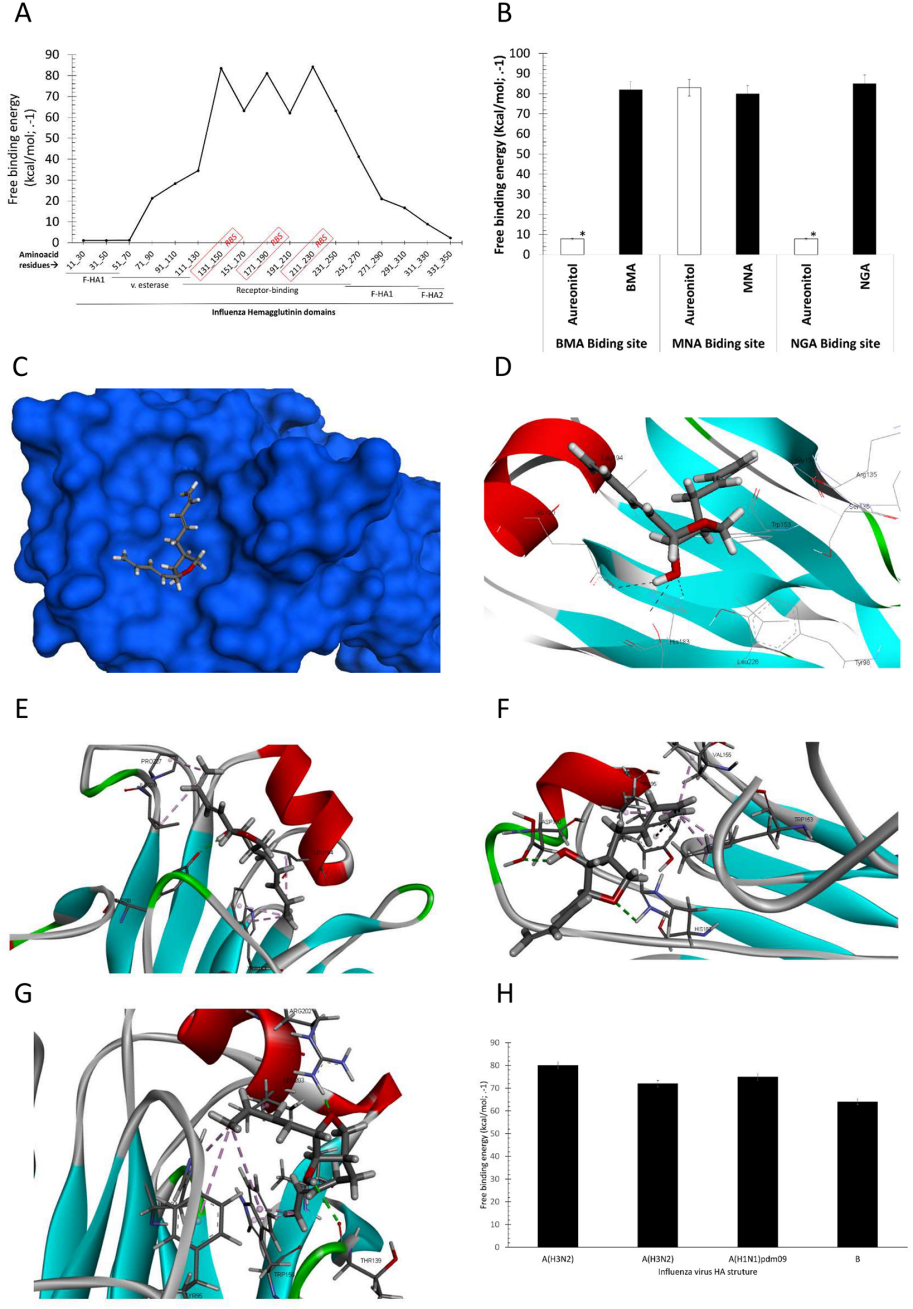
*In silico* analysis of aureonitol docked to influenza HA. (A) Aureonitol was docked over all 19 amino acid residues of influenza A(H3N2) HA (pdb #1HGE). (B) Aureonitol was docked on the BMA, MNA and NGA binding sites, using these molecules as a reference. (C) Crystal structure of the H3 isoform mentioned above (blue) and aureonitol (gray) docked in the globular head of HA, the sialic acid binding site. Aureonitol interacts with conserved amino acid residues by hydrogen and Pi-Alkalyl bonds with HAs representative of laboratory-adapted influenza A(H3N2) (D) and clinically isolated influenza A(H3N2) (E), influenza A(H1N1)pdm09 (F) and Influenza B (G). Free binding energies were calculated for the interactions between aureonitol and each HA structure used in panels D through G (H). Docking was performed three times. *P < 0.01 indicates significant differences.

To gain insight on the aureonitol docking site, we performed *in silico* studies. We evaluated the entire influenza HA structure by docking aureonitol at each of the 19 amino acid residues to better comprehend the most likely binding site. We observed that the free binding energies were lower in the sialic acid binding site, also known as the receptor binding site (RBS) in the three-dimensional receptor binding domain (Fig 5A). Surprisingly, aureonitol binding was versatile, showing higher free energies for other areas of the influenza HA (Fig 5A). To confirm aureonitol docking in the RBS, its binding was specifically evaluated in the binding sites of 2-O-methyl-5-N-acetyl-α-D-neuraminic acid (MNA), β-D-manose (BMA), and N-acetyl-D-glucosamine (NGA). MNA is docked in the RBS, whereas BMA and NGA were used as negative controls. As shown in Fig 5B, the free binding energy was lower for aureonitol`s docking at the MNA binding site, indicating that our compound nestled most spontaneously in this site (Fig 5C). Aureonitol`s docking site is located in HA`s globular head in an area that is rich in amino acid residues that are conserved for most of influenza strains[48, 49]. Aureonitol interacted by hydrogen bonding with the amino acids Tyr98, His183, Glu190 and Ser228 (Fig 5D and Table E in S1 File), which are critical for HA-cellular receptor interactions during influenza entry [49].

Because the HA structure used to bind aureonitol is derived from the laboratory-adapted strain of influenza A(H3N2) used in our study, we also evaluated aureonitol docking in the HA structures of other influenza viruses. Interestingly, aureonitol docked in the influenza A(H3N2) HA, a viral structure that is closer to the clinically isolated strain used in our investigation, with the polymorphism E190D (Fig 5E and Table E in S1 File) and used the amino acid residues mentioned in the paragraph above, except for the Glu190. For influenza A(H1N1)pdm09, aureonitol formed hydrogen bonds with the polymorphic residue Asp190 and Gln226, in addition to the residues mentioned in the first structure (Fig 5F and Table E in S1 File). With influenza B HA, aureonitol formed fewer hydrogen bonds, targeting the Arg202 and Thr139 residues (Fig 5G and Table E in S1 File). In addition to hydrogen bonds, other weak interactions were also observed between aureonitol and the HAs analyzed (Table E in S1 File). The free binding energies to dock aureonitol in the different HAs were proportional to the number of hydrogen bonds formed (Fig 5H and Table E in S1 File), indicating that aureonitol binds more spontaneously to influenza A(H3N2) and A(H1N1)pdm09 than to influenza B. These results predict the most likely binding site of aureonitol in the HA structure and highlight that this molecule is a promising candidate for the development of novel anti-influenza drugs.

## Discussion

Influenza viruses cause a recurrent public health problem for populations who are at higher risk for serious illness, such as newborns, the elderly, pregnant women and immunocompromised individuals [1–3], and constantly impose threats of pandemic outbreaks[1–3]. Antiviral drugs against influenza are essential to fight both seasonal and pandemic infections[1–3]. Because influenza antivirals are more effective when administered within the first days after the onset of illness[50], stockpiling of anti-influenza drugs that can be used as the first line of defense against a novel virus is a key step in pandemic preparedness. Moreover, considering the emergence of drug-resistant strains of influenza, the search for molecules that are able to inhibit the influenza life cycle at different steps than molecules currently in clinical use is pivotal. Here, we show that aureonitol, a fungi-derived natural product, inhibits influenza attachment/entry by targeting conserved amino acid residues on the viral surface glycoprotein HA.

Aureonitol inhibited influenza replication at nanomolar concentrations and with very low cytotoxicity. Consequently, aureonitol presented a very safe range to be used *in vitro*. Our compound was approximately 100-times more potent than other fungi-derived natural products previously studied against influenza[51, 52]. Aureonitol’s chemical structure has a THF ring, and other molecules with this same ring have shown antiviral activity [23–26], including against influenza NA. Notably, under our experimental conditions, no anti-NA activity of aureonitol was been observed.

When compared to other influenza inhibitors that are in clinical use, such as OST, aureonitol was slightly less potent. Nevertheless, OST and aureonitol possess different mechanisms of action. Whereas OST blocks the spread of influenza by inhibiting the viral enzyme NA, aureonitol impairs influenza entry by targeting the viral HA. Importantly, circulating strains of influenza are resistant to adamantanes, and approximately 2% are resistant to OST [11, 12]. Thus, the effect of aureonitol on a different step of the virus life cycle may be of great interest. Other investigators have previously attempted to block virus entry by targeting influenza HA. An example is the promising new compound Flufirvitide-3, which is currently being tested in clinical trials [53]. Another molecule, arbidol, which is an indole ring with substituents in almost all positions, also inhibits influenza replication by targeting the viral HA [54]. Although arbidol is not approved for clinical use in western countries, it is being used clinically in Russia and China [45, 54, 55].

Aureonitol binds in versatile ways to influenza HA at the sialic acid binding site in the RBD. This pocket in the HA structure is responsible for binding the sialic acid residues on cell surfaces for influenza entry [56]. Therefore, the sialic acids binding site is extremely conserved in HA, even in influenza strains carrying mutated HA that escapes the host immune response. In HA’s sialic acid binding site, the conserved amino acid residues His183, Tyr98 and Glu190 have polar side chains and interact with the receptor by hydrogen bonding, while Trp153, Leu194 and Leu226 have non-polar side chains that facilitate receptor binding through Van Der Waals interactions [56]. Our results suggest that aureonitol disrupts these interactions between influenza HA and sialic acid, which could result in a much weaker interface between the virus and cell surface, thereby preventing viral entry. Considering that the conserved amino acid residues in influenza HA are responsible for viral entry along with the molecular modeling results for aureonitol, it is possible that influenza strains resistant to our compound might lose fitness/virulence. This possibility is currently under investigation in our laboratories.

Since natural products are a fruitful source of chemically rich compounds, our laboratory has been studying the antiviral activity of these molecules [28, 57–60]. In this article, we pay special attention to the antiviral activity of fungi-derived natural products, which may have been overlooked in the literature. Interestingly, fungi members of the genus *Chaetomium* have been isolated from traditional medicinal plants from China and India and are recognized to be the organisms producing the bioactive compounds [61, 62]. Aureonitol is produced by different species of the genus *Chaetomium* [63]. When compared to other natural products, aureonitol possesses some advantageous features. Different species of *Chaetomium* produce aureonitol with over 70% yield[63], and organic synthesis of aureonitol has be proven to be successful [21]. Because these characteristics indicate the feasibility of scaling up aureonitol production and our result show that this molecule inhibits influenza replication by targeting viral entry via conserved residues on HA, aureonitol’s chemical structure may be of interest for further development of anti-influenza drugs.

## Supporting Information

S1 File Includes all the supporting Information, Tables A-E and Figs A-H and their legends. (RAR)
