# Correction: Aureonitol, a Fungi-Derived Tetrahydrofuran, Inhibits Influenza Replication by Targeting Its Surface Glycoprotein Hemagglutinin

**DOI:** 10.1371/journal.pone.0142246

**Published:** 2015-10-30

**Authors:** Carolina Q. Sacramento, Andressa Marttorelli, Natalia Fintelman-Rodrigues, Caroline S. de Freitas, Gabrielle R. de Melo, Marco E. N. Rocha, Carlos R. Kaiser, Katia F. Rodrigues, Gisela L. da Costa, Cristiane M. Alves, Osvaldo Santos-Filho, Jussara P. Barbosa, Thiago Moreno L. Souza

There is an error in affiliation 3 for authors Carolina Q. Sacramento, Andressa Marttorelli, Natalia Fintelman-Rodrigues, Caroline S. de Freitas, Gabrielle R. de Melo, Marco E. N. Rocha, and Thiago Moreno L. Souza. Affiliation 3 should be: National Institute for Science and Technology on Innovation on Neglected Diseases (INCT/IDN), Center for Technological Development in Health (CDTS), Oswaldo Cruz Foundation (Fiocruz), Rio de Janeiro, RJ, Brazil.
